# Clinical predictors of outcomes in patients undergoing emergency air medical transport from Kinmen to Taiwan

**DOI:** 10.1097/MD.0000000000008440

**Published:** 2017-11-03

**Authors:** Julia Chia-Yu Chang, Hsien-Hao Huang, Shu-Hua Chang, Yin-Ru Chen, Ju-Shin Fan, Yen-Chia Chen, David Hung-Tsang Yen

**Affiliations:** a Department of Emergency Medicine, Taipei Veterans General Hospital; b Institute of Emergency and Critical Care Medicine, College of Medicine, National Yang-Ming University, Taipei; c Department of Nursing, Taipei Veterans General Hospital, Taiwan, R.O.C.

**Keywords:** emergency air medical transport, emergency department, intensive care

## Abstract

Emergency air medical transport (EAMT) is indispensable for acutely or critically ill patients in remote areas. We determined patient-level and transport-specific factors associated with all-cause mortality after EAMT.

We conducted a population-based, retrospective cohort study using a prospective registry consisting of clinical/medical records. Study inclusion criteria consisted of all adults undergoing EAMT from Kinmen hospital to the ED of Taipei Veterans General Hospital (TVGH) between January 1, 2006 and December 31, 2012. The primary outcome assessments were 7-day and 30-day mortality.

A total of 370 patients transported to TVGH were enrolled in the study with a mean age of 54.5 ± 21.5 (SD) years and with a male predominance (71.6%). The average in-transit time was 1.4 ± 0.4 hours. The 7-day, 30-day, and in-hospital mortality rates were 10.3%, 14.1%, and 14.9%. Among them 33.5% (124/370) were categorized under neurological etiologies, whereas 24.9% (90/370) cardiovascular, followed by 16.2% (60/370) trauma patients. Independent predictors associated with 7-day all-cause mortality were age (odds ratio [OR] 1.043, 95% confidence interval [CI] 1.016–1.070), Glasgow Coma Scale (GCS) (OR 0.730, 95% CI 0.650–0.821), and hematocrit level (OR 0.930, 95% CI 0.878–0.985). Independent predictors associated with 30-day all-cause mortality were age (OR 1.028, 95% CI 1.007–1.049), GCS (OR 0.686, 95% CI 0.600–0.785), hematocrit (OR 0.940, 95% CI 0.895–0.988), hemodynamic instability (OR 5.088 95% CI 1.769–14.635), and endotracheal intubation (OR 0.131 95% CI 0.030–0.569). The 7-day and 30-day mortality were not significantly related to transport-specific factors, such as length of flight, type of paramedic crew on board, or day and season of transport. Clinical patient-level factors, as opposed to transport-level factors, were associated with 7- and 30-day all-cause mortality in patients undergoing interfacility EAMT from Kinmen to Taiwan.

## 1 Introduction

Transport of acutely injured and ill patients by air has become an integral part of the regionalized system of health care. Patient outcomes are improved with the use of trauma systems that allow emergency air medical transport (EAMT) of seriously injured patients from remote locations to the nearest appropriate trauma center.^[1–5]^ EAMT has a beneficial impact in terms of mortality in patients sustaining severe blunt trauma.^[2]^ Nontrauma patients such as those with an ST elevation myocardial infarction^[6,7]^ or acute stroke,^[8,9]^ requiring time-critical intervention, also benefit with improved outcomes. EAMT proves to be a safe and feasible mean of transportation in head injury^[10,11]^ obstetric,^[12]^ neonatal,^[13]^ and toxicological^[14]^ emergencies.

EAMT is an inseparable part of health care in Kinmen County, an island 120 km away from Taiwan. The geographical barrier separating Taiwan from its remote islands promotes growing needs for EAMT,^[15]^ which operates under policy and regulations set by the National Aeromedical Approval Center (NAAC) established in 2002 for better alignment of emergency resources.

Despite the growing need for EAMT, debates persist regarding the benefit, cost-effectiveness, safety, and risk of air transport. Mortality after EMAT is an important indicator to evaluate effectiveness of transport and the acute management of critically ill patients. This study was conducted to determine both patient-level and transport-specific characteristics that were associated with 7-day and 30-day mortality after EMAT.

## 2 Methods

### 2.1 Study design

**Figure 1:**
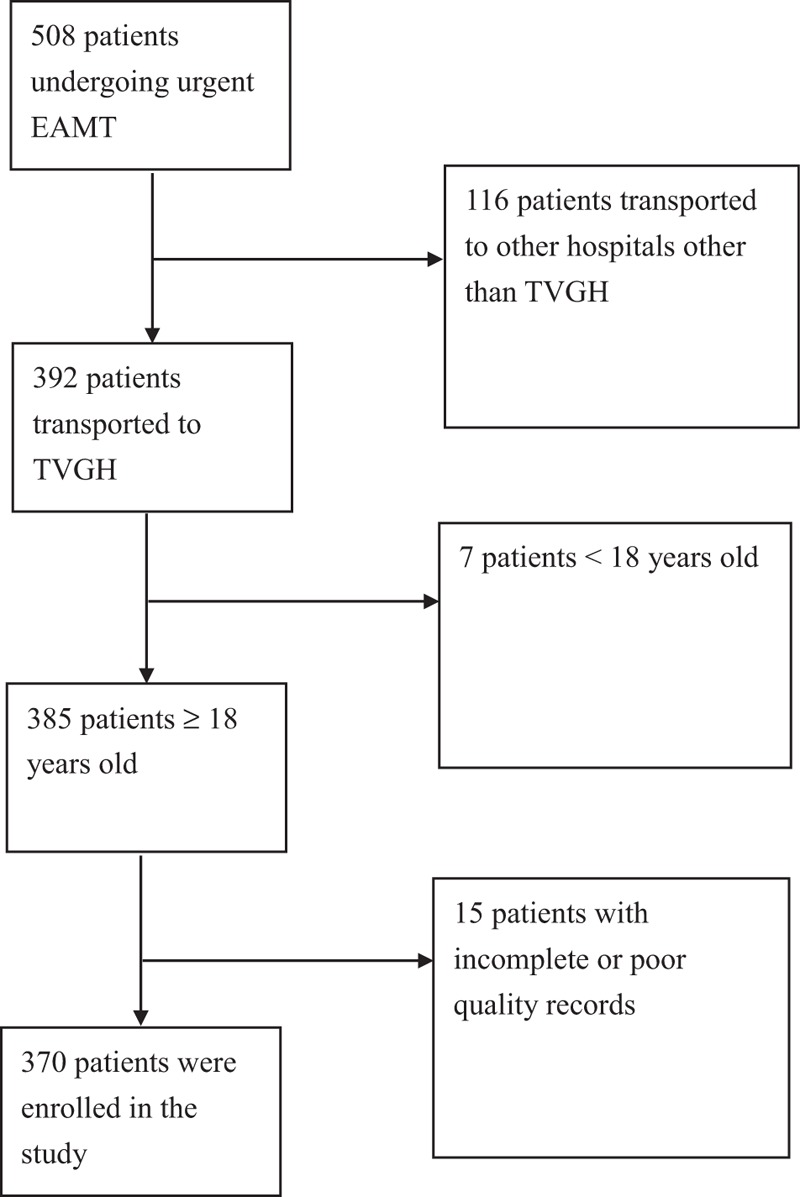
Flow chart illustrating the selection of patients. EAMT = emergency air medical transport, TVGH = Taipei Veterans General Hospital.

We conducted a retrospective cohort study on EAMT from Kinmen hospital to the ED of TVGH from January 1, 2006 to December 31, 2012 in which all adults undergoing EAMT from Kinmen to the ED of TVGH were enrolled. The major exclusion criteria were age <18 years, death during transport, nonurgent transports, and transport to other hospitals other than TVGH (Fig. 1). A final of 370 patients were enrolled in the study. EAMT, under specified criteria^[16]^ for appropriate patient selection, was conducted using rotary-wing (helicopter) aircrafts with specially trained paramedic flight crew along with either an in-flight nursing staff, emergency medical technician-paramedic (EMT-P), or a transport medical physician to the ED of TVGH, a 3000-bed, university-affiliated medical center. Clinical data were recorded in-flight by the transport crew, and medical records were accessible in TVGH. The hospital's institutional review board approved this study with a waived patient informed consent.

### 2.2 Data collection

The eligibility criteria were checked by 2 emergency physicians at TVGH. Missing, aberrant. and illogical data were excluded. We obtained data on the clinical and demographic characteristics of patients and transport-specific characteristic from the in-flight and hospital records. The clinical characteristics of all the study patients, including triage categories using Taiwan triage and acuity scale (TTAS),^[17]^ Glasgow Coma Scale (GCS), basic laboratory work consisted of hemogram, biochemical tests, and arterial blood gas, traumatic or nontraumatic classifications, time of arrival at the emergency department (ED), ED length of stay (LOS), administration of assisted ventilation, need for emergency operation or time-critical interventions, admission to intensive care unit (ICU) or ordinary wards, time and day of arrival to the ED, and length of flight, were collected from either the medical records or the electronic database system at TVGH. TTAS, a 5-level Taiwan triage system modified from Canadian triage and acuity scale (CTAS) with permission from the CTAS national working group. TTAS has been validated as a reliable triage system that accurately prioritize treatments and avoid overtriage in the ED.^[17]^ We defined baseline hemodynamic instability as a systolic blood pressure of <80 mmHg or a mean arterial pressure of <60 mmHg before departure from Kinmen hospital, or the administration of vasopressors before departure from Kinmen hospital and upon arrival to the TVGH ED.

Transport-specific characteristics obtained included: seasons, days (weekends vs. weekdays) and time (day vs. night) of transport, types of transport crew and time (day vs. night), and day (weekends vs. weekdays) of admissions. The transports and admissions were considered taking place during daytime if it occurred between 8:00 am and 5:00 pm. Weekends are defined between 8:00 am on Saturday until 8:00 am on Monday.

### 2.3 Outcome measures

The primary outcomes of this study were all-cause mortality within 7 and 30 days of air medical transport. The secondary outcome is in-hospital all-cause mortality.

### 2.4 Data analysis

Data are expressed as mean ± standard deviation (SD) for continuous variables and as number (%) for categorical variables. The categorical variables were compared by using the *χ*^2^ or Fisher exact test. The distribution of data was assessed with the Kolmogorov–Smirnov test. Comparisons of numerical variables were performed using an unpaired *t* test (parametric data) or Mann–Whitney *U* test (nonparametric data). Statistical analysis was performed using SPSS software version 15.0 (SPSS Inc, Chicago, IL). Statistical tests were 2-sided, and the significance level was set at *P* < .05. Variables with *P* < .1 in the univariate analysis were further analyzed for outcome assessment by using multivariate regression analysis

## 3 Results

During the study period, we identified 370 patients meeting the study criteria (Fig. 1). The mean age was 54.5 years, and 71.6% of the patients were male (Table 1). The average time of flight from Kinmen to Taipei was 1.4 ± 0.4 hours. Among the patients, a majority of them (85.1%) were accompanied by either a nursing staff or an EMT-P, whereas 14.9% by medical physicians. More than half (62.4%) of the flights were made during the night (from 5 pm to 8 am the next day) and 71.1% were made during weekdays. More than half (65.9%) of the patients were admitted during the night, and 70.7% were admitted during weekdays. The proportions of flight numbers during spring, summer, and winter were 25.1%, 27.6%, and 26.8%, respectively, with a slight decrease in flight number in autumn (20.5%). Upon arrival to the ED at TVGH, the mean GCS score was 11.4 ± 4.6, which did not deteriorate as compared to the GCS in the local hospital (11.9 ± 4.6) before EAMT. Among the transferred patients, 59.8% were triaged under Taiwan triage and acuity scale (TTAS) category 1 and 33.7% under TTAS category 2 at the receiving hospital. Overall, 44.6% were admitted to the ICU, 42.7% received emergent interventional treatments, either surgery or catheterization, 9.5% were admitted to the ward, 5 patients (1.4%) were directly discharged from the ED, whereas 7 patients (1.9%) expired in the ED. The overall 7-day, 30-day, and in-hospital all-cause mortality rates were 10.3%, 14.1%, and 14.9%, respectively.

**Table 1:**
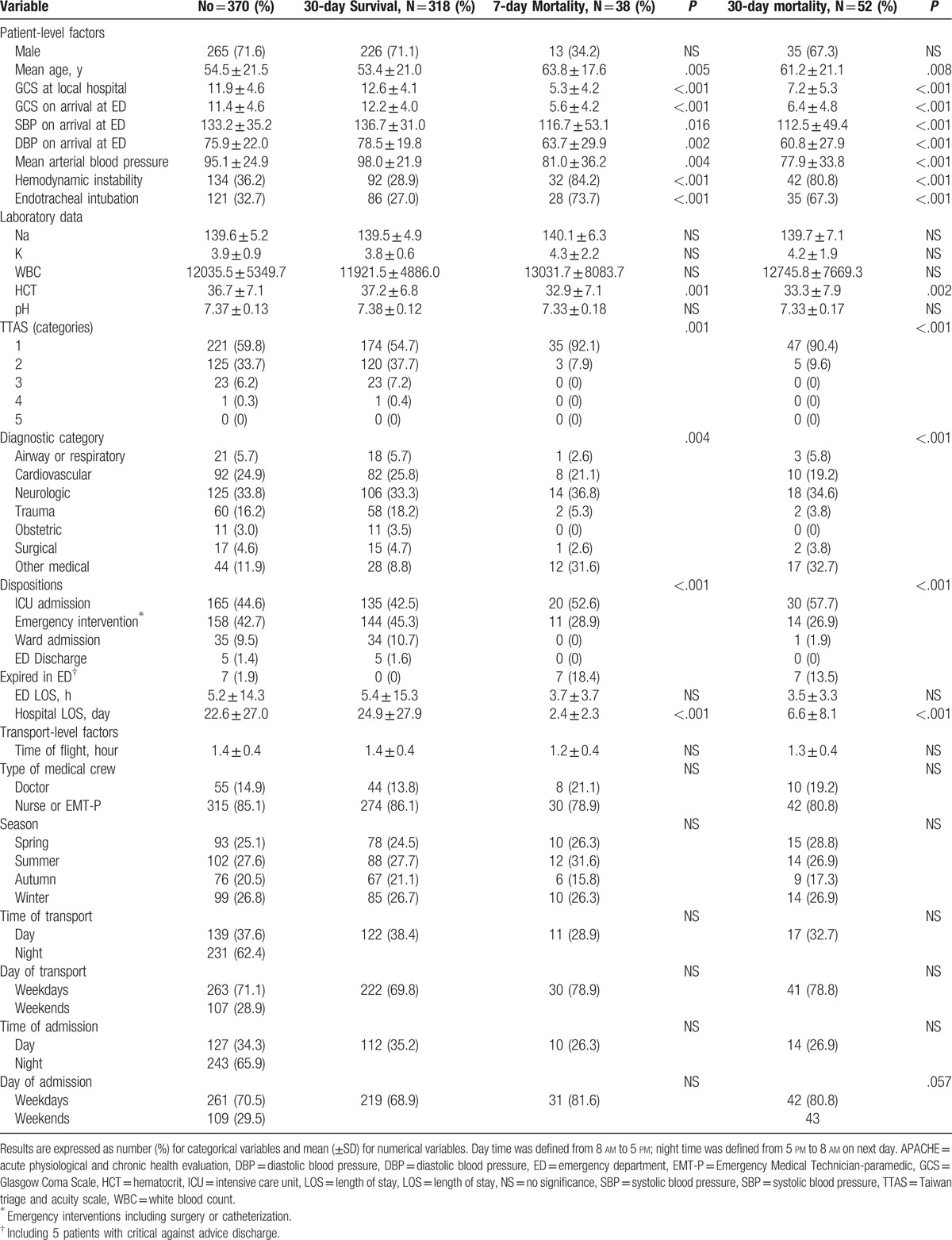
Comparison of patient-level and transport-level characteristics between 30-day survival and 7-day and 30-day all-cause mortality.

Among these transferred patients, 33.8% were categorized under neurological etiologies, including intracranial or subdural hematoma requiring neurosurgical consultation, emergent operation, or stroke requiring intensive care admission (Table 2), whereas 24.9% were under cardiovascular etiologies, including acute myocardial infarction or unstable angina requiring either catheterization or cardiac unit care. Trauma patients requiring surgical consultation and definitive treatments attributed to 16.2% of the EAMT. Airway or respiratory diseases with or without endotracheal intubation contributed to 5.7% of the transfers, whereas surgical cases 4.6%, obstetric 3.0%, and miscellaneous cases 11.8%. The percentage of patients transferred for trauma and surgical etiology that received surgical interventions were 58.3% (35/60) and 58.8% (10/17), respectively. Nearly half (48.0% [60/125]) of patients with neurological etiology received surgical intervention, whereas 42.3% (39/92) of patients with cardiovascular etiology received catheterization.

**Table 2:**
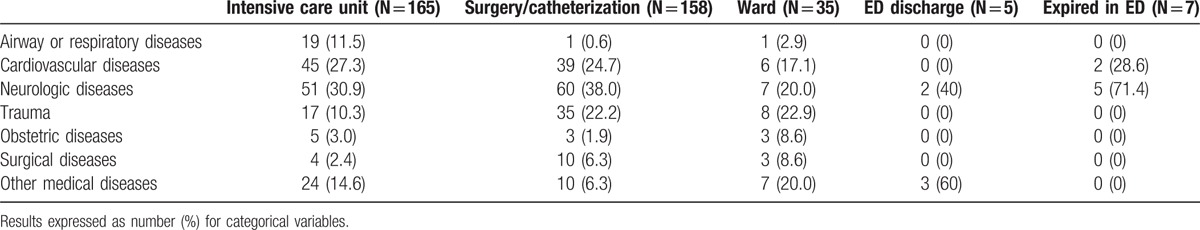
Diagnostic categories and dispositions of 370 patients at the referral hospital.

Several patient-level characteristics identified were associated with 7-day and 30-day all-cause mortality (*P* < .05): patient age, GCS, hemodynamic instability, mean blood pressure, endotracheal intubation, hematocrit level, and disease categories. Transfer-level factors such as type of medical crew, seasons, time and day of transport, and time and day of admission were not associated with 7-day and 30-day mortality (Table 1). Diagnostic categories were associated with differences in outcome. Patients transferred because of trauma and obstetric etiology had better outcome than other categories with a 30-day survival of 96.6% (58/60) and 100% (11/11), respectively. The 30-day survival in patients transferred for cardiovascular, surgical, airway/respiratory, and neurologic etiology was 89.1% (82/92), 88.2% (15/17), 85.7% (18/21), and 84.8% (106/125), respectively.

Patient-level characteristics such age (odds ratio [OR] 1.043), Glasgow Coma Scale (GCS) (OR 0.730), and hematocrit level (OR 0.930) were independent predictors for 7-day all-cause mortality (*P* < .05) (Table 3.). Patient-level characteristics of age (OR 1.028), GCS (OR 0.686), hematocrit (OR 0.940), hemodynamic instability (OR 5.088), and endotracheal intubation (OR 0.131) were independent predictors for 30-day all-cause mortality (*P* < .05).

**Table 3:**
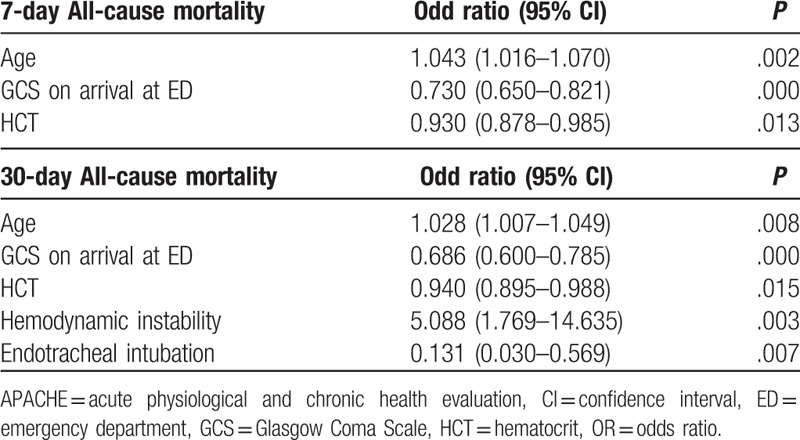
Multiple logistic regression backward analysis of clinical characteristics associated with 7-day and 30-days all-cause mortality.

## 4 Discussion

The study found several patient-level characteristics as opposed to transport-level factors associated with both 7-day and 30-day all-cause mortality in patients undergoing EAMT from Kinmen to TVGH. Patient-level characteristics of age, consciousness (GCS), and hematocrit level were independently associated with both 7-day and 30-day all-cause mortality. Additional patient-level characteristics of hemodynamic instability and endotracheal intubation status were independently associated with 30-day mortality. Age was positively correlated whereas higher GCS and higher hematocrit levels were negatively correlated with both 7- and 30-day mortality. This finding suggested older patients, patients with lower GCS, or lower hematocrit levels had higher risks for short- and long-term all-cause mortality after EAMT. Hemodynamic instability was positively correlated, whereas endotracheal intubation negatively correlated with 30-day mortality. It is interesting to note that patients’ hemodynamic instability was not reflected immediately in 7-day mortality after EAMT; however, it was reflected in patients’ long-term outcome in 30-day mortality. The study found patients who were not endotracheal intubated had a higher risk for 30-day mortality after EAMT. This demonstrated whether patients’ condition mandated intubation and intubation was not performed for any reason, may it be the signing of do not resuscitate (DNR) agreement owing to old age, or terminal illnesses, the patient had a higher risk for 30-day all-cause mortality after EAMT. Our study did not investigate the number of DNR agreements signed before nor after EAMT. However, this did leave room for discussion on futile treatment, patient selection, benefit, and whether EAMT was truly justified in these cases.

Our study demonstrates age as a significant factor associated with both 7-day and 30-day all-cause mortality, which is in accordance with previous studies.^[15,18–20]^ Whether EAMT benefits the elderly, as it does the young population is yet to be determined. However, with an increase in aging population, an increase in EAMT for the elderly is to be expected. In fact, Chen et al^[15]^ discovered the average age of patient who underwent EAMT increased during 2006 to 2011 and the elderly comprised the majority (56.6%) of mortality cases. Age should be a consideration in appropriate patient selection for EAMT, as it is a risk factor associated with mortality. Appropriate criteria for patient selection would serve to determine when EAMT is justified and best benefit patients.

Different diagnostic categories were associated with differences in outcomes. Patients transferred because of trauma and obstetric etiology had better outcome than other categories with 30-day survival of 96.6% (58/60) and 100% (11/11), respectively. Previous reports also demonstrated the beneficial impact of EAMT on mortality in severe blunt trauma^[2]^ and seriously injured patient.^[3,4,21,22]^ The utilization of EAMT for patients with serious traumatic injuries may be expensive, but it is justified by its cost-effectiveness^[23]^ and the low in-hospital mortality observed in our study. Our study also concurred with the Japanese study by Ohara et al on the safety and usefulness of emergency maternal transport using helicopter.^[12]^ A total of 125 patients (33.8%) were transferred owing to neurological conditions, which concurred with Chen et al^[24]^ who demonstrated the predominance of neurological diseases (47% of all patients) among Taiwanese patients transported via international aeromedical services.

Several transport-level factors may hinder and delay rapid access to EAMT, including weather conditions, seasons of the year, day of the week, availability of transportation vehicles, and even patient's weight.^[25]^ In this study, transport-level factors were not significantly associated with 7- and 30-day all-cause mortality. Our result demonstrates that patients’ outcomes depended less on transport-specific details, but rather more on patient-level characteristics. However, Singh et al^[26]^ demonstrated that both patient- and transport-level factors were independently associated with in-transit critical events. Further prospective study should be conducted to investigate how patient-level and transport-level factors influence in-transit critical events and whether in-transit critical events influence mortality after EAMT.

## 5 Limitations

This study has numerous limitations. First, as a retrospective study it was subject to incomplete or missing data either at local hospital, during transit or at referral hospital. Second, this study was carried out in a single referral medical center. The results may not be generalizable to other settings with different magnitude or nonmedical contract hospitals. Therefore, it was not possible to compare similar patients transported and treated by other hospitals. However, during the study period, 392 of the 508 patients (77.16%) were transferred to TVGH via EAMT, representing a majority of population undergoing this EAMT system. Third, although mortality is an important endpoint, our study did not incorporate other important patient outcomes such as functional capacity at discharge. Lastly, the lack of in-transit critical events observed during study period may be because of poor quality in-transit records or the capacity of flight crew and the difficulty to perform invasive procedures such as endotracheal intubation, needle thoracostomy or cricothyroidotomy during flight. Hence, discussion on in-transit critical events was beyond the scope of our study. Despite these limitations, there were relatively few potential confounders in this study because it was conducted within the same patient settings, homogeneous cooperative dispatch system, and a single referral medical center.

## 6 Conclusions

The study identified several clinical patient-level characteristics as opposed to transport-level factors associated with both 7-day and 30-day all-cause mortality in patients undergoing EAMT from Kinmen to TVGH. Predictive parameters of age, GCS, hematocrit level, hemodynamics, and endotracheal intubation status should be taken into consideration in risk assessment before EAMT, and for short- and long-term outcome evaluation after EAMT. These findings serve as a reference for better implementation of EAMT within the integrated delivery care system and for future modification of EAMT criteria for appropriate patient selection.
